# Chloroquine Dosing Recommendations for Pediatric COVID‐19 Supported by Modeling and Simulation

**DOI:** 10.1002/cpt.1864

**Published:** 2020-05-21

**Authors:** Laurens F. M. Verscheijden, Tjitske M. van der Zanden, Lianne P. M. van Bussel, Marika de Hoop‐Sommen, Frans G. M. Russel, Trevor N. Johnson, Saskia N. de Wildt

**Affiliations:** ^1^ Department of Pharmacology and Toxicology Radboud Institute for Molecular Life Sciences Radboud University Medical Center Nijmegen The Netherlands; ^2^ Dutch Knowledge Center Pharmacotherapy for Children The Hague The Netherlands; ^3^ Department of Paediatrics Erasmus MC Sophia Children's Hospital Rotterdam The Netherlands; ^4^ Royal Dutch Pharmacist Association The Hague The Netherlands; ^5^ Certara UK Limited Sheffield UK; ^6^ Intensive Care and Department of Paediatrics Surgery Erasmus MC‐Sophia Children's Hospital Rotterdam The Netherlands

## Abstract

As chloroquine (CHQ) is part of the Dutch Centre for Infectious Disease Control coronavirus disease 2019 (COVID‐19) experimental treatment guideline, pediatric dosing guidelines are needed. Recent pediatric data suggest that existing World Health Organization (WHO) dosing guidelines for children with malaria are suboptimal. The aim of our study was to establish best‐evidence to inform pediatric CHQ doses for children infected with COVID‐19. A previously developed physiologically‐based pharmacokinetic (PBPK) model for CHQ was used to simulate exposure in adults and children and verified against published pharmacokinetic data. The COVID‐19 recommended adult dosage regimen of 44 mg/kg total was tested in adults and children to evaluate the extent of variation in exposure. Based on differences in area under the concentration‐time curve from zero to 70 hours (AUC_0–70h_) the optimal CHQ dose was determined in children of different ages compared with adults. Revised doses were re‐introduced into the model to verify that overall CHQ exposure in each age band was within 5% of the predicted adult value. Simulations showed differences in drug exposure in children of different ages and adults when the same body‐weight based dose is given. As such, we propose the following total cumulative doses: 35 mg/kg (CHQ base) for children 0–1 month, 47 mg/kg for 1–6 months, 55 mg/kg for 6 months–12 years, and 44 mg/kg for adolescents and adults, not to exceed 3,300 mg in any patient. Our study supports age‐adjusted CHQ dosing in children with COVID‐19 in order to avoid suboptimal or toxic doses. The knowledge‐driven, model‐informed dose selection paradigm can serve as a science‐based alternative to recommend pediatric dosing when pediatric clinical trial data is absent.

**Study Highlights**

**WHAT IS THE CURRENT KNOWLEDGE ON THE TOPIC?**

☑ Chloroquine (CHQ) is proposed for coronavirus disease 2019 (COVID‐19) treatment. CHQ for malaria treatment in children is safe, but pediatric dose requirements for COVID‐19 are lacking.

**WHAT QUESTION DID THIS STUDY ADDRESS?**

☑ What is the best‐evidence, model‐informed CHQ dose to recommend when pediatric clinical trial data is absent?

**WHAT DOES THIS STUDY ADD TO OUR KNOWLEDGE?**

☑ Pediatric CHQ dose recommendations for COVID‐19 were determined. Children 6 months to 12 years of age are in need of 30% higher mg/kg doses, whereas younger children need 20% lower doses than older children and adults.

**HOW MIGHT THIS CHANGE CLINICAL PHARMACOLOGY OR TRANSLATIONAL SCIENCE?**

☑ A knowledge driven, model‐informed approach can be used to support off‐label dose recommendations, in the absence of clinical data, in case of a high medical need.

With coronavirus disease 2019 (COVID‐19) spreading rapidly across the globe, effective drug treatment is desperately needed. Based on Chinese population data, < 1% of the infected cases were children below the age of 10 years and clinical symptoms in these patients were milder compared with adults.^1^ However, it is unknown how such numbers will evolve when a higher percentage of the entire world population with diverse ethnic and socioeconomical backgrounds is infected.

Emerging data prompted the US Food and Drug Administration (FDA) to issue the emergency use authorization of chloroquine (CHQ) to treat COVID‐19.^2^ The mechanism of action of CHQ for COVID‐19 is not known, but it has been hypothesized that CHQ acts through inhibition of endosome‐mediated viral entry, and pH dependent steps in viral replication.^3^ In addition, reduced cytokine release by immune cells could possibly benefit patients having a severe immune response.^3^ For adults, *in vitro* and modeling studies have explored the potentially effective plasma and tissue concentrations to treat COVID‐19.^4^, ^5^ The Dutch Centre for Infectious Disease Control (CIDC) recommends a total cumulative dose of 3,300 mg CHQ base (44 mg/kg for a 75 kg adult), reducing the risk of adverse events by limiting treatment to 5 days.^6^ The Dutch Pediatric Formulary (DPF), the government supported, national source for pediatric drug information and drug doses,^7^ set out to determine a best‐evidence CHQ dose for children with COVID‐19.

As CHQ is licensed for use in children with malaria,^8^ it may seem rational to use the World Health Organization (WHO) recommended antimalarial dose to treat COVID‐19 infected pediatric patients (25 mg/kg given over 3 days).^9^ However, applying these licensed doses may not be optimal to treat COVID‐19. In fact, the current pediatric WHO dose may even be questionable for the treatment of malaria, as recent studies show that older infants and children may need a higher mg/kg dose to reach similar drug concentrations as adults.^10^, ^11^, ^12^ In contrast, it is likely that neonates and young infants will need lower doses per kg body weight. CHQ is metabolized by the drug metabolizing enzymes CYP3A4 (~ 15%) and CYP2C8 (~ 20%) and is renally excreted (~ 56%, leaving ~ 9% unknown additional clearance).^4^ All these processes are immature at birth and show an increase to adult values in the first years of life.^13^

Physiologically‐based pharmacokinetic (PBPK) modeling combines drug‐specific properties and physiological properties to model drug disposition and drug action. Both the FDA as well as the European Medicine Agency support the use of these models to determine the optimal dose also for children.^14^, ^15^, ^16^

Hence, we aim to model and simulate CHQ exposure in children and propose optimal dosing regimens for COVID‐19.

## Methods

### PBPK model building

Simcyp version 19 was used for simulations, which were performed using the predefined North European white “Sim‐Healthy volunteer” and “Sim‐Paediatric” populations. Default age‐related physiological parameters (e.g., ontogeny in CYP3A4 expression, CYP2C8 expression, and renal clearance) were verified previously.^17^, ^18^ Parameters in pediatric subjects were redefined over time as explained by Abduljalil *et al.* ^19^ The CHQ compound file, built and verified by Yao *et al.* in adults, was derived from the Simcyp repository and used without modifications.^4^ For all simulations, the proportion of female patients was set to 0.5.

### PBPK model verification

To verify the model, plasma CHQ concentrations from Zhao *et al*. (American, healthy adults, African, malaria‐infected children) and Karunajeewa *et al.* (Melanesian, malaria‐infected children) were compared with the simulated concentrations.^10^, ^20^ Adults received a single 300 mg CHQ base tablet, the children (6 months–12 years) ~ 10 mg base/kg (cut) tablets q.d. for 3 days, respectively. Only minimum and maximum concentrations were extracted from the published figures using WebPlotDigitizer version 4.1, because individual values could not be identified from the plots.

Simulations were performed for at least 400 individuals per age group and trial size was based on the number of patients in the studies above. For adults, a single dose of 300 mg was simulated for 10 trials of 40 subjects (18–65 years).

For children, doses of 10 mg/kg q.d. for 3 days (30 mg/kg total) were simulated. For the 6‐month to 5‐year‐olds, 4 trials of 123 subjects were simulated, and for the 5 to 12‐year‐olds, 6 trials of 76 subjects. Simulations were run for 7 days and clinically measured data were overlaid with PBPK simulated concentration‐time profiles to verify the CHQ PBPK model.

### Dose selection in children

Simulations were performed using the standard body weight (75 kg) normalized adult oral dose of 44 mg base/kg, as proposed by the Dutch CIDC.^6^ A loading doses of 8 mg/kg is followed by 4 mg/kg after 12 hours. Maintenance doses on days 2–5 are given as 4 mg/kg twice daily. Age groups were defined as 0–1 month, 1–6 months, 6 months to 5 years, 5–12 years, and adults (18–65 years) based on the age groups defined in the Zhao *et al.* PK study (3 oldest cohorts), and expected age‐related changes based on CYP and GFR maturation (2 youngest cohorts). Simulations were performed in 10 trials using 40 subjects per trial and run over 70‐day periods. The area under the curve (AUC) was calculated from 0 up until ~ 5 reported half‐lives (70 days) after the first dose, at which point virtually all drug is removed from the system.^8^ AUC_adult_ and AUC_pediatric_ values were used to optimize dosing regimens by multiplying the AUC ratio with total adult dose (44 mg/kg) using this formula:

(1) $$\text{Total pediatric dose}=\frac{\text{AUCadult}}{\text{AUCpediatric}}*\text{Total adult dose}$$

The loading doses tested were the licensed pediatric dose (10 mg/kg followed after 6 hours by 5 mg/kg) and the Dutch CIDC adult dose (8 mg/kg followed after 12 hours by 4 mg/kg). The remainder of the total pediatric dose was equally divided over 8 doses administered twice daily on days 2–5. Revised doses were then re‐entered into the simulator to confirm that they resulted in similar exposure (within a 5% limit) compared with the adult dose. We assume that matching systemic CHQ exposures in the different age groups results in a similar lung exposure, as predicted by Yao *et al*.

## Results

### Model verification

**Figure 1:**
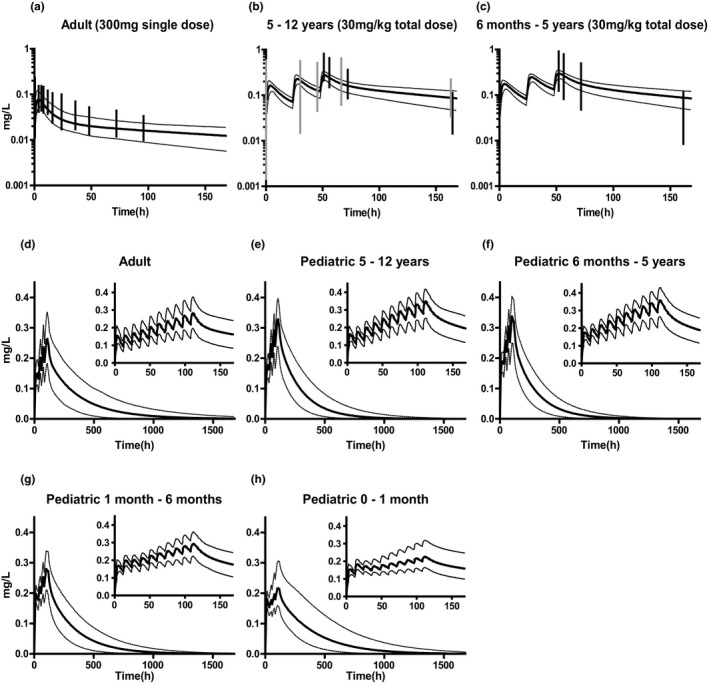
Simulations of chloroquine (CHQ) pharmacokinetic (PK) profiles for model verification (**a–c**) and individual age‐group adjusted CHQ PK predictions (**d–h**). Simulations of CHQ concentration‐time profiles in **a** adults (300 mg, single dose) and **b**, **c** children 6 months to 12 years (10 mg/kg q.d. for 3 days). Solid lines indicate mean simulated values. Dotted lines indicate the first and 99th simulated percentile. Black vertical lines indicate the range between minimum and maximum values reported by Zhao *et al*.^10^ Grey vertical lines indicate the range between minimum and maximum values reported by Karunajeewa *et al*.^20^ Age adjusted doses: (**d**) Adult simulation. Dose on day 1: 8 mg/kg followed by 4 mg/kg after 12 hours. Dose on days 2–5: 4 mg/kg twice daily. (**e**) Pediatric simulation in children 5–12 years of age. Dose on day 1: 10 mg/kg followed by 5 mg/kg after 12 hours. Dose on days 2–5: 5.4 mg/kg twice daily. (**f**) Pediatric simulation in children 6 months to 5 years of age. Dose on day 1: 10 mg/kg followed by 5 mg/kg after 12 hours. Dose on days 2–5: 5.2 mg/kg twice daily. (**g**) Pediatric simulation in children 1–6 months of age. Dose on day 1: 10 mg/kg followed by 5 mg/kg after 12 hours. Dose on days 2–5: 3.7 mg/kg twice daily. (**h**) Pediatric simulation in children 0–1 month of age. Dose on day 1: 10 mg/kg followed by 5 mg/kg after 12 hours. Dose on days 2–5: 2.5 mg/kg twice daily. Panels in the right upper corner indicate simulations in the first week of treatment.

PBPK model simulations were compared with observed values in adults and children 6 months to 12 years of age (**Figure** **1a–c**). Mean plasma concentrations were predicted well, as our doses fell within the reported concentrations ranges. Around 160 hours after dosing, the model slightly overpredicted measured concentrations by Zhao *et al*. Observed variability seemed larger than predicted variability.

### Dose selection in children

Simulations with the recommended adult dose (44 mg/kg) in all age groups resulted in AUC ratios (adult/pediatric age group) diverting from 1 and differing by age group (**Table** **1**). Using these AUC ratios, new dosing regimens were calculated and simulated for the previously defined age groups (**Figure** **1d–h**). The new age‐adjusted doses, with corresponding AUC values are reported in **Table** **1**. To simplify the dosing schedule for clinical use, the dose recommendations were rounded (**Table** **1**).

**Table 1:** Age‐adjusted pediatric dose recommendation

Age group	AUC ratio (AUC_adult_/ AUC_pediatric_)	New age adjusted dose (mg/kg)	AUC after new age adjusted dose (SEM) mg/L.hour	Final age‐adjusted dose advice (mg/kg)
Adults and children > 12 years	NA	44 (day 1: 8 mg/kg, followed by 4 mg/kg after 12 hours. Days 2–5: 4 mg/kg twice daily).	76.6 (1.1)	44 mg/kg^a^ (day 1: 8 mg/kg, followed by 4 mg/kg after 12 hours. Days 2–5: 4 mg/kg twice daily; max dose: 3,300 mg).
Pediatric 5–12 years	1.33	58.5 (day 1: 10 mg/kg, followed by 5 mg/kg after 12 hours. Days 2–5: 5.4 mg/kg twice daily).	76.4 (0.8)	55 mg/kg (day 1: 10 mg/kg, followed by 5 mg/kg after 12 hours. Days 2–5: 5 mg/kg twice daily; max dose: 3,300 mg).
Pediatric 6 months to 5 years	1.29	56.8 (day 1: 10 mg/kg, followed by 5 mg/kg after 12 hours. Days 2–5: 5.2 mg/kg twice daily).	76.1 (0.8)	55 mg/kg (day 1: 10 mg/kg, followed by 5 mg/kg after 12 hours. Days 2–5: 5 mg/kg twice daily).
Pediatric 1–6 months	1.01	44.4 (day 1: 10 mg/kg, followed by 5 mg/kg after 12 hours. Days 2–5: 3.7 mg/kg twice daily).	76.9 (0.8)	47 mg/kg (day 1: 10 mg/kg, followed by 5 mg/kg after 12 hours. Days 2–5: 4 mg/kg twice daily).
Pediatric 0–1 month	0.80	35.2 (day 1: 10 mg/kg, followed by 5 mg/kg after 12 hours. Days 2–5: 2.5 mg/kg twice daily).	77.3 (1.1)	35 mg/kg (day 1: 10 mg/kg, followed by 5 mg/kg after 12 hours. Days 2–5: 2.5 mg/kg twice daily).

AUC, area under the curve; NA, not applicable.

^a^ Adults receive a dose for an average (75 kg) individual irrespective of body weight (44 mg/kg × 75 kg = 3,300 mg total dose).

## Discussion

Our study presents age‐adjusted CHQ doses for treatment of COVID‐19 in children across the pediatric age range. These doses support previous pharmacokinetic (PK) studies in pediatric patients with malaria showing a need for higher mg/kg doses, as compared with adults, to reach similar plasma exposures in children 6 months to 12 years.^10^, ^11^, ^12^ In addition, these doses account for immature drug metabolism and renal function, as reflected by the need of lower doses in children < 6 months of age, compared with older children.^13^

We verified our model by comparing simulated and observed concentrations from published PK studies in adults and children. Mean concentration‐time profiles seemed to reasonably reflect the observed CHQ concentrations.^10^, ^20^ The simulated variability tended to be smaller than that observed in children. This may be explained by rounding of the dose in the original studies, as the oral tablet formulation did not allow precise weight‐based dosing. In one study, tablets were cut into quarters and halves, in the other study, two strength tablets were used.^10^, ^20^ As simulations were in the same range as the published data and generally captured central tendency of these data, the model is considered adequate for the purpose of pediatric dose extrapolation based on exposure‐matching.

As the dosing schedule proposes higher mg/kg doses than the currently licensed pediatric dose for malaria treatment, safety questions may arise. Especially as lethal toxicity in children has been reported.^21^, ^22^ The children in these toxicity reports received unintentional overdoses of ~ 35–100 mg/kg CHQ (base) in a single dose.^21^ These doses are much higher than proposed here. In studies in children > 6 months with malaria, doses of 50 and 70 mg/kg (in a 3‐day schedule) were well‐tolerated, with no severe cardiac adverse event.^11^, ^23^ For the youngest age groups, exposure matching should result in similar concentrations as in older children. From literature search and consultancy with malaria experts, no special safety issues for neonates emerged, only after significant overdoses. In general, children with QT prolongation had very low mortality.^24^ Nevertheless, we recommend daily monitoring of potential adverse events, including corrected QT interval prolongation by echocardiogram monitoring, before treatment and daily afterward.

Our study has several other limitations and assumptions. We used a dosing schedule aiming for similar plasma exposures as with the adult dose proposed by the Dutch CIDC.^6^ This is a pragmatic combination of the registered malaria dose and the prolonged treatment suggested by the recent COVID‐19 studies, avoiding higher than adult exposures. If currently ongoing studies show more optimal dosing regimens in adults for COVID‐19, we will reconsider pediatric doses using the same concept of exposure‐matching informed by PBPK modeling.

In addition, the plasma target for COVID‐19 is not clearly established. The reported *in vitro* antiviral half‐maximal effective concentrations of CHQ (half‐maximal effective concentration = 5.47 μM ref. 4 and 1.13 μM ref. 5) will not be reached in plasma by the current proposed dose (factor 2–10 too low). Considering the extremely large volume of distribution of CHQ (100–200 L/kg^20^), up to 400 times higher concentrations may be reached in lung tissue, providing adequate antiviral exposure, as simulated by Yao *et al*.^4^ In this study, we assume that matching systemic exposure in adults would result in similar lung concentration of CHQ in pediatrics, which requires further investigation.

Another limitation of our model is the lack of PK data to verify the pediatric CHQ simulations of children < 6 months of age. The SIMCYP pediatric model has been extensively verified for CYP3A, CYP2C8, and renally cleared drugs in this age range.^17^, ^18^ Moreover, the CHQ dose predictions are in line with our understanding of maturation of these processes.^13^ Regarding absorption, the compound file developed by Yao *et al*. uses a first‐order absorption model developed using adult data only.^4^ As absorption in adults is almost complete, we do not expect a major effect of this limitation on the plasma concentrations in children.

If efficacy for treatment of COVID‐19 is to be evaluated in children, it is important that the right dose will be used, leading to comparable exposures as in adults. As the expected number of COVID‐19 pediatric patients in need of CHQ treatment will be small, randomized trials to evaluate efficacy are less feasible in this population. Collecting sparse PK data, especially in neonates and infants under CHQ treatment for COVID‐19, for whom currently no data are available, allows further verification and improvement of the PBPK model. In addition, although ethically challenging, data on tissue distribution will be extremely valuable to confirm predictive performance of future models.

In conclusion, we present best‐evidence CHQ doses for pediatric COVID‐19. We recommend the use of these doses to provide children optimal exposure with the highest chance of efficacy and safety. The knowledge‐driven, model‐informed dose selection paradigm presented in this study can serve as a science‐based alternative for the DPF to recommend pediatric dosing when pediatric clinical trial data is absent.

## Funding

This work was supported by the Bill & Melinda Gates Foundation (INV 001822).

## Conflict of Interest

T.N.J. is an employee of Certara UK Limited and involved in the development of the commercial Simcyp PBPK model. T.M.Z. is technical director of the Dutch Paediatric Pharmacotherapy Expertise Network. S.N.W. is medical director of Dutch Paediatric Pharmacotherapy Expertise Network. All other authors declared no competing interests for this work.

## Author Contributions

All authors wrote the manuscript. S.N.W. designed the research. L.F.M.V, T.M.Z., L.P.M.B., and M.H.S. performed the research. All authors analyzed the data.
